# Reply to D. Bush et al

**DOI:** 10.1200/GO.23.00402

**Published:** 2024-01-25

**Authors:** Desmond Yip, Andrew Soma, Nalini Pati, Wendy Spencer, Beth Hua, Rooney Jagilly

**Affiliations:** Desmond Yip, MBBS, FRACP, Department of Medical Oncology, The Canberra Hospital, Garran, ACT, Australia, ANU Medical School, Australian National University, Canberra, ACT, Australia; Andrew Soma, MBBS, NRH Oncology Unit, National Referral Hospital, Honiara, Solomon Islands; Nalini Pati, MBBS, MD, DNB, DCHFRACP, FRCPA, ANU Medical School, Australian National University, Canberra, ACT, Australia, Department of Haematology, The Canberra Hospital, Garran, ACT, Australia; Wendy Spencer, RN, Medical Day Treatment Unit, The Canberra Hospital, Garran ACT, Australia; Beth Hua, BPharm, GCertS, MBA, Department of Health and Aged Care, Australian Government, Canberra, Australia; and Rooney Jagilly, MBBS, MMed (Surg), Department of General Surgery, National Referral Hospital, Honiara, Solomon Islands

We thank Bush et al^1^ for their correspondence enquiring about the outcomes and sustainability of the National Referral Hospital (NRH) oncology unit.^2^

The recognition by the hospital executive of the unit, allocation of resources by increase in staff from one doctor to two and two nursing staff to five (all full time equivalent positions) since inception (thus enabling backfill of leave), commensurate with the growth in level of activity, and endorsement of and support of a formal training pathway for the nation's first oncologist shows the government's commitment to sustain the unit in the long term. Encouragingly, there are ongoing initiatives to establish an in-patient cancer ward at the NRH for both adults and children and to expand palliative care services, further attesting to the commitment to advancing cancer care.

In the Solomon Islands, the National Medical Stores (NMS) is responsible for the central management and distribution of health commodities, and it remains the responsibility of the Solomon Island Ministry of Health and Medical Services (MHMS) at the government level. Over the past few years (before the COVID-19 pandemic), there were preexisting local impacts to the workflow of the NMS at the government level. Sudden and unplanned shifts made to their procurement processes created a rapid decline in the availability of essential medicines and medical supplies in 2018. The COVID-19 pandemic provided a further shock to the supply chains in the Pacific region.

Supply chain challenges in global health care are not exclusive to low-resource nations. Indeed the issue of medication availability constitutes a worldwide concern that compounds the intrinsic system issues within individual countries, thereby significantly influencing the ongoing treatment of oncology patients. This has still been affecting both developed and low-resource countries even after the pandemic.

A recent Solomon Islands Health Supply Chain Assessment and Recommendations report prepared by an independent organization for the Solomon Islands MHMS and the Australian Department of Foreign Affairs and Trade (DFAT), with the support of the Indo-Pacific Center for Health Security, details an end-to-end assessment of supply chain in the country. The report contains a review of the Solomon Islands health supply chain current state, integrated with detailed recommendations for strengthening and reform.

A change in management and support from key stakeholders such as independent organizations working with the Solomon Island MHMS and multiple ministries are assisting to facilitate positive changes. It remains the remit of the Solomon Island Government to continue to manage their processes. However targeted technical assistance from Australia after identification of the key issues has provided them with a more supported and transparent approach forward.

We agree that success cannot be purely judged on caseload. The NRH oncology unit keeps a database of patients treated in the unit. Because of the wide geographical spread of the patients' places of residence, it has been challenging to collect follow-up information to determine relapse or death. Although appointments are made at 1 month, 3 months, 6 months, and then annually after completion of curative or adjuvant therapy, not all patients will attend because of these geographical and socioeconomic reasons. The hospital-based Solomon Islands Cancer Registry has also had significant technical problems in the past 2-3 years, and data on new registrations collected on hard copy forms have not been able to be entered. It has faced the same difficulties in collecting survival information. Ideally comparative data should be available from population-based cancer registries to be able to determine the effect of treatment interventions.

This does highlight the importance for ongoing international support and collaboration in ensuring the continuity of medication supply, as well as implementation of reliable databases that actively record incidence cases of cancers and outcome information to demonstrate the benefit of therapies.

## References

[1] Bush D, Love M, Bugoro H, et al: The Solomon Islands oncology unit: Sustainability in terms of outcomes. JCO Glob Oncol 10.1200/GO.23.00325 10.1200/GO.23.00325 PMC10830091 38271650

[2] Yip D, Soma A, Pati N, et al: Building capability and capacity: The establishment of an oncology unit in the Solomon Islands. JCO Glob Oncol 10.1200/GO.22.00325 10.1200/GO.22.00325 PMC10497292 36862976

